# Health workers’ adoption of digital health technology in low- and middle-income countries: a systematic review and meta-analysis

**DOI:** 10.2471/BLT.24.292157

**Published:** 2024-12-03

**Authors:** Minmin Wang, Kepei Huang, Xiangning Li, Xuetong Zhao, Laura Downey, Sondus Hassounah, Xiaoyun Liu, Yinzi Jin, Minghui Ren

**Affiliations:** a Department of Global Health, School of Public Health, Peking University, 38 Xue Yuan Road, Haidian District, Beijing 100191, China.; b School of Public Health, Imperial College London, London, England.; c School of Public Health, Peking University, Beijing, China.; d George Institute for Global Health, University of New South Wales, Sydney, Australia.; e China Center for Health Development Studies, Peking University, Beijing, China.; Correspondence to Yinzi Jin (email: yzjin@bjmu.edu.cn).

## Abstract

**Objective:**

To conduct a systematic review and meta-analysis of the facilitators of and barriers to the acceptance and use of digital health technology by health workers in low- and middle-income countries.

**Methods:**

We searched several databases for relevant articles published until 25 April 2024. We extracted data on four unified theories of acceptance and use of technology factors (performance expectancy, effort expectancy, social influence and facilitating conditions) and six additional factors (attitude, habit, incentive, risk, trust and self-efficacy); how these affected the outcomes of behavioural intention and actual use; and the strength of association if reported. We conducted a meta-analysis of the quantitative studies.

**Findings:**

We reviewed 36 publications, 20 of which were included in our meta-analysis. We observed that performance expectancy was the most frequently reported facilitator (in 21 studies; 58.3%) and that lack of facilitating conditions was the most cited barrier (10; 27.8%). From our meta-analysis, trust (*r* = 0.53; 95% confidence interval, CI: 0.18 to 0.76) and facilitating conditions (*r* = 0.42; 95% CI: 0.27 to 0.55) were the leading facilitators of behavioural intention and actual use, respectively. We identified concerns with performance expectancy (*r* = −0.14, 95% CI: −0.29 to 0.01) as the primary barrier to both outcomes.

**Conclusion:**

Our approach of clustering the facilitators of and barriers to the acceptance and use of digital health technology from the perspective of health workers highlighted the importance of creating an enabling ecosystem. Supportive infrastructure, tailored training programmes and incentive policies should be incorporated in the implementation of digital health programmes in low- and middle-income countries.

## Résumé

**Objectif:**

Réaliser une revue systématique et une méta-analyse des facteurs facilitants et des obstacles à l’acceptation et à l’utilisation des technologies numériques médicales par les professionnels de la santé de pays à revenu faible et intermédiaire.

**Méthodes:**

Nous avons consulté plusieurs bases de données pour trouver des articles pertinents publiés jusqu’au 25 avril 2024. Nous avons extrait des données sur: quatre facteurs de la théorie unifiée de l’acceptation et de l’utilisation de la technologie (attentes de performance, attentes d’effort, influence sociale et conditions facilitantes) ainsi que six facteurs supplémentaires (attitude, habitude, incitation, risque, confiance et auto-efficacité); la façon dont ces facteurs ont affecté les résultats de l’intention comportementale et de l’utilisation effective; et la force de l’association si elle était mentionnée. Nous avons réalisé une méta-analyse des études quantitatives.

**Résultats:**

Nous avons examiné 36 publications et en avons inclus 20 à notre méta-analyse. Nous avons observé que les attentes de performance étaient le facteur facilitant le plus souvent mentionné (dans 21 études, 58,3%) et que les conditions facilitantes étaient l’obstacle le plus souvent cité (dans 10 études, 27,8%). D’après notre méta-analyse, la confiance (*r *= 0,53; intervalle de confiance (IC) à 95%: 0,18 à 0,76) et les conditions facilitantes (*r* = 0,42; IC à 95%: 0,27 à 0,55) constituaient les principaux facteurs facilitants de l’intention comportementale et de l’utilisation effective, respectivement. Nous avons identifié des problèmes liés aux attentes de performance (*r* = -0, 14, IC à 95%: -0,29 à 0,01) comme le principal obstacle à ces deux résultats.

**Conclusion:**

Notre approche consistant à regrouper les facteurs facilitants et les obstacles à l’acceptation et à l’utilisation des technologies numériques médicales du point de vue des professionnels de la santé a mis en évidence l’importance de la création d’un écosystème propice. Des infrastructures de soutien, des programmes de formation adaptés et des politiques d’incitation doivent s’intégrer dans la mise en œuvre des programmes de santé numériques dans les pays à revenu faible ou intermédiaire.

## Resumen

**Objetivo:**

Realizar una revisión sistemática y un metaanálisis de los factores que facilitan y dificultan la aceptación y el uso de la tecnología sanitaria digital por parte de los agentes de salud en los países de ingresos bajos y medios.

**Métodos:**

Se realizaron búsquedas de artículos relevantes publicados hasta el 25 de abril de 2024 en varias bases de datos. Se extrajeron datos sobre: cuatro factores de la teoría unificada de la aceptación y el uso de la tecnología (expectativa de rendimiento, expectativa de esfuerzo, influencia social y condiciones facilitadoras) y seis factores adicionales (actitud, hábito, incentivo, riesgo, confianza y autoeficacia); cómo afectaban a los resultados de la intención de comportamiento y el uso real; y la fuerza de la asociación si se informaba de ella. Se realizó un metaanálisis de los estudios cuantitativos.

**Resultados:**

Se revisaron 36 publicaciones, 20 de las cuales se incluyeron en el metaanálisis. Se observó que la expectativa de rendimiento era el facilitador más mencionado (en 21 estudios; 58,3%) y que las condiciones facilitadoras eran el obstáculo más citado (10; 27,8%). Según el metaanálisis, la confianza (*r* = 0,53; intervalo de confianza del 95%: 0,18 a 0,76) y las condiciones favorables (*r* = 0,42; IC del 95%: 0,27 a 0,55) fueron los principales facilitadores de la intención de comportamiento y el uso real, respectivamente. Se identificó la preocupación por las expectativas de rendimiento (*r* = -0, 14; IC del 95%: -0,29 a 0,01) como el principal obstáculo para ambos resultados.

**Conclusión:**

El planteamiento de agrupar los factores que facilitan y dificultan la aceptación y el uso de la tecnología sanitaria digital desde la perspectiva de los agentes de salud destacó la importancia de crear un entorno propicio. La infraestructura de apoyo, los programas de formación personalizados y las políticas de incentivos deben incorporarse a la implementación de programas de salud digital en países de ingresos bajos y medios.

## ملخص

**الغرض:**

إجراء مراجعة منهجية وتحليل تلوي للعوامل المساعدة والعوائق أمام قبول واستخدام تكنولوجيا الصحة الرقمية بواسطة العاملين في القطاع الصحي في الدول ذات الدخل المنخفضة والدخل المتوسط.

**الطريقة:**

بحثنا في العديد من قواعد البيانات عن المقالات ذات الصلة المنشورة حتى 25 أبريل/نيسان 2024. قمنا باستخراج البيانات عن: أربع نظريات موحدة لقبول العوامل التكنولوجية واستخدامها (توقع الأداء، وتوقع الجهد، والنفوذ الاجتماعي، والظروف المساعدة)، وستة عوامل إضافية (الموقف، والعادة، والحافز، والمخاطرة، والثقة، والفعالية الذاتية)؛ وكيف أثرت هذه العوامل على نتائج النية السلوكية والاستخدام الفعلي؛ وقوة الارتباط إذا تم الإبلاغ عنها. وقم بإجراء تحليل تلوي للدراسات الكمية.

**النتائج:**

قمنا بمراجعة 36 منشورًا، تم تضمين 20 منها في تحليلنا التلوي. لاحظنا أن توقع الأداء كانت العامل المساعد الأكثر شيوعًا (في 21 دراسة؛ بنسبة %58.3)، وأن الظروف المساعدة كانت الحاجز الذي تم الاستشهاد به بشكل أكثر (10؛ بنسبة %27.8). ومن خلال تحليلنا التلوي، كانت الثقة ( *r * = 0.53؛ بفاصل ثقة مقداره %95: 0.18 إلى 0.76)، والظروف المساعدة ( *r * = 0.42؛ بفاصل ثقة مقداره %95: 0.27 إلى 0.55) العاملان المساعدان الرئيسيان للنية السلوكية والاستخدام الفعلي، على التوالي. قمنا بتحديد المخاوف بشأن توقع الأداء ( *r *= -0.14، بفاصل ثقة مقداره %95: -0.29 إلى 0.01) باعتبارها الحاجز الأساسي لكلا النتيجتين.

**الاستنتاج:**

إن أسلوبنا في تجميع العوامل المساعدة والحواجز أمام قبول واستخدام تكنولوجيا الصحة الرقمية من منظور العاملين في القطاع الصحي، قد قام بالتركيز على أهمية إنشاء نظام بيئي داعم. ويجب دمج البنية الأساسية الداعمة، وبرامج التدريب المخصصة، وسياسات الحوافز، في تنفيذ برامج الصحة الرقمية في الدول ذات الدخل المنخفض والدخل المتوسط.

## 摘要

**目的:**

对促进和阻碍中低收入国家卫生工作者接受和使用数字卫生技术的因素进行系统评价和荟萃分析。

**方法:**

我们在多个数据库中检索了截至 2024 年 4 月 25 日发表的相关文章。我们就以下几个方面进行了数据提取：接受和使用技术的四个统一理论因素（绩效期望、付出期望、社会影响和促进条件）和六个额外因素（态度、习惯、激励、风险、信任和自我效能）；这些因素如何影响行为意向和实际使用的结果；以及关联强度（如有报告）。我们对定量研究进行了荟萃分析。

**结果:**

我们审查了 36 篇出版物，其中 20 篇被纳入了我们的荟萃分析。据我们观察，绩效期望是报告中被提及频率最高的促进因素（在 21 项研究中有提及；占 58.3%），而促进条件是被提及频率最高的阻碍因素（在 10 研究中有提及；占 27.8%）。从我们的荟萃分析结果来看，信任（*r* = 0.53；95% 置信区间 (CI)：0.18 至 0.76）和促进条件（*r* = 0.42；95% CI：0.27 至 0.55）分别为促进行为意向和实际使用的最主要因素。我们发现，对绩效期望的担忧（*r* =  –0.14，95% CI：–0.29 至 0.01）是阻碍达成两种结果的主要因素。

**结论:**

我们从卫生工作者的角度对促进和阻碍接受和使用数字卫生技术的因素进行了分类，所用方法强调了创建有利生态系统的重要性。在中低收入国家实施数字健康计划时，应考虑配套基础设施以及定制培训计划和激励政策。

## Резюме

**Цель:**

Провести систематический обзор и метаанализ факторов, способствующих и препятствующих принятию и использованию цифровых технологий в сфере здравоохранения медицинскими работниками в странах с низким и средним уровнем дохода.

**Методы:**

Был проведен поиск соответствующих статей, опубликованных до 25 апреля 2024 года, в нескольких базах данных. В результате было получено описание четырех факторов единой теории принятия и использования технологий (ожидание результатов, ожидание усилий, социальное влияние и благоприятные условия), шести дополнительных факторов (отношение, привычка, стимул, риск, доверие и уверенность в собственных силах), того, как они повлияли на результаты поведенческих намерений и фактического использования, а также силы связи, если таковая была выявлена. Был проведен метаанализ количественных исследований.

**Результаты:**

Был проведен анализ 36 публикаций, 20 из которых были включены в метаанализ. Согласно полученным данным, ожидание результатов было наиболее часто упоминаемым фактором содействия (в 21 исследовании; 58,3%), а наиболее часто упоминаемым препятствием были благоприятные условия (10 исследований; 27,8%). По данным проведенного авторами метаанализа, доверие (*r* = 0,53; 95%-й ДИ: 0,18–0,76) и благоприятные условия (*r* = 0,42; 95%-й ДИ: 0,27–0,55) были ведущими факторами, способствующими поведенческому намерению и фактическому использованию соответственно. Основным препятствием для достижения обоих результатов являются проблемы с ожиданием результатов (*r* = –0,14, 95%-й ДИ: от –0,29 до 0,01).

**Вывод:**

Разработанный подход к группировке факторов, способствующих и препятствующих принятию и использованию цифровых технологий здравоохранения с точки зрения медицинских работников, подчеркнул важность создания благоприятной экосистемы. При реализации программ цифрового здравоохранения в странах с низким и средним уровнем дохода необходимо предусмотреть вспомогательную инфраструктуру, специализированные программы обучения и политику стимулирования.

## Introduction

Digital health technology can make health systems more efficient and sustainable, facilitating the provision of high-quality care across a wide range of contexts and for diverse population health needs. The pace of innovation in digital health is rapid and constant, with new interventions being developed, implemented, tested and refined against a diversity of contexts, constraints and challenges to address a variety of health and health system needs. These evolving capabilities in technology are being routinely leveraged as interventions within digital applications to aid individuals, the health workforce and health system users in improving access, coverage, equity and quality of health services.^1^^,^^2^ However, the implementation of digital health technology remains unsatisfactory,^3^^,^^4^ and the facilitators of and barriers to implementation have been largely understudied, particularly in low- and middle-income countries; such a research gap contributes to the digital divide and related health inequity between countries of lower and higher incomes.

The potential for digital health technology to transform health-care utilization and delivery has been recognized for over two decades. Through its 2005 resolution WHA58.28 on electronic health (eHealth), the World Health Assembly urged Member States “to consider drawing up a long-term strategic plan for developing and implementing eHealth services, to develop the infrastructure for information and communication technologies for health, and to promote equitable, affordable, and universal access to the benefits of eHealth.”^5^ In 2021, the World Health Assembly endorsed the establishment of the World Health Organization’s *Global strategy on digital health 2020–2025*.^6^ This strategy is based on four principles and requires that countries decide and commit to digital innovation; recognize that successful digital technologies require an integrated strategy; promote the appropriate use of digital interventions for health; and address the major impediments faced by the least developed countries implementing digital health technology. 

Despite the existence of global strategies and calls for action, research on facilitators of and barriers to the acceptance and use of digital health technology in low- and middle-income countries is fragmented and sparse, especially with regards to the viewpoint of health workers. We therefore conducted a systematic review and meta-analysis to address these gaps in the literature, and determine the factors that drive or impede the adoption of digital health technology by health workers in low- and middle-income countries.

## Methods

We registered our study with the International Prospective Register of Systematic Reviews (CRD42024559814), and conducted our systematic review and meta-analysis in line with the preferred reporting items for systematic review and meta-analyses guidelines.^7^

### Data sources and searches

We searched the databases PubMed®, Embase®, Web of Science, Latin American and Caribbean Health Sciences Literature, China National Knowledge Infrastructure and WanFang Database from inception to 25 April 2024. We used medical subject headings (MeSH) and free-text identifiers associated with digital health, technology acceptance, framework and low- and middle-income countries. We provide the detailed literature search strategy in our online repository.^8^ Three authors independently screened the titles and abstracts of retrieved citations to identify relevant studies, and then independently performed the full-text evaluations of the selected articles. We resolved any disagreements by consensus.

### Study selection and quality 

We considered studies to be eligible for inclusion if they reported facilitators of and barriers to the acceptance and use of digital health technology by health workers in low- and middle-income countries. We included randomized controlled trials, and observational, cross-sectional or cohort studies published in peer-reviewed journals. We excluded case studies, conference papers, systematic reviews, meta-analyses or bibliometrics. We excluded publications that (i) reported on studies conducted in high-income countries; (ii) only reported the effectiveness of digital health technology without exploring the factors influencing its acceptance; or (iii) focused on the viewpoints of patients or the community as opposed to health workers. We included qualitative, quantitative and mixed-method studies, and did not apply any language restrictions. We used the translation tool DeepL Translate (DeepL SE, Cologne, Germany) to assist with our understanding of articles published in languages other than English or Chinese. 

Two authors independently assessed the methodological quality and risk of bias of included studies by applying the recommendations of the United States Agency for Healthcare Research and Quality (AHRQ).^9^ The AHRQ score is calculated from 11 quality indicators; a score of 0–4, 5–7 or 8–11 indicates a high, moderate or low risk of bias, respectively.

### Data extraction and synthesis 

We evaluated and collated findings using an adapted version of a thematic synthesis.^10^ We applied the unified theory of acceptance and use of technology framework to categorize the facilitators and barriers influencing the acceptance and use of digital health technologies. The framework synthesizes several related innovation adoption theories^11^^,^^12^ to include four main domains: performance expectancy, the degree to which an individual believes that using the system will enhance job performance; effort expectancy, the perceived degree of ease associated with the use of the system; social influence, how the beliefs of others that the system should be adopted are considered; and facilitating conditions, the degree to which an individual believes that an organizational and technical infrastructure exists to support the use of the system. To these four domains, we added six further domains of attitude, habit, incentive, risk, trust and self-efficacy. 

Two authors extracted data from each study, including general study information such as study design, sample size and country; reported facilitators of and barriers to the acceptance and use of digital health technology by health workers (categorized in terms of the 10 factor domains); the effect of these factors on one of two possible outcomes (behavioural intention and actual use); where relevant, the effect of behavioural intention on actual use; and, for quantitative studies, the strength of any association (e.g. Pearson correlation coefficients) reported for any given factor. 

We calculated the frequency of occurrence for 21 different paths: the 20 paths from categorized facilitator or barrier to associated outcome; and, because some studies also described how behavioural intention affected actual use, the path from behavioural intention to actual use.

### Meta-analysis

To estimate the strengths of the facilitators and barriers in the framework domains, we conducted a meta-analysis of the studies that reported Pearson correlation coefficients (or other statistics that could be converted to correlation coefficients by structural equation modelling). For factors identified as both facilitators and barriers, we conducted separate meta-analyses according to their effect. We tested heterogeneity across studies by performing Cochrane’s *Q* test and the *I*^2^ index.^13^ We calculated the correlation coefficient (*r*) with 95% confidence interval (CI) for each path using a random effect model. We generated funnel plots to determine the existence of potential publication bias. Additionally, we performed Begg rank correlation and Egger linear regression tests to determine publication bias, with *P*-value less than 0.05 indicating significant publication bias.^14^^,^^15^ We conducted subgroup analyses to further evaluate the potential heterogeneity between upper-middle-income countries and lower-middle- and low-income countries. Finally, we also conducted sensitivity analyses by only including studies with a low or moderate risk of bias.

We conducted all statistical analyses for this study using R software, version 4.1.3 (R Core Team, Vienna, Austria). All tests were two-sided, and *P*-values less than 0.05 were considered statistically significant.

## Results

### Study selection and characteristics

**Fig. 1:**
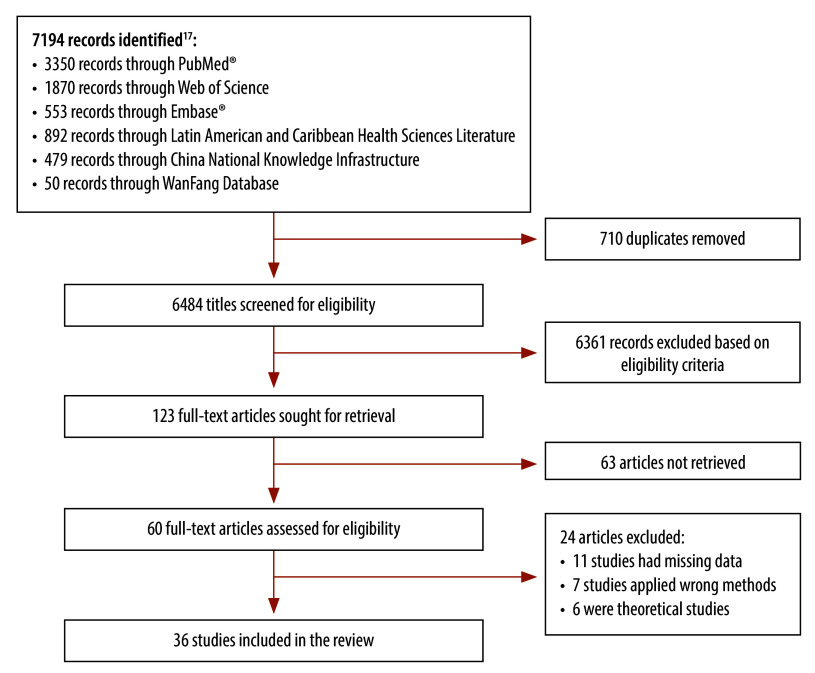
Flowchart of the selection of studies on acceptance and use of digital health technology by health workers in low- and middle-income countries

Our search yielded a total of 7194 records across all accessed databases. After removal of duplicates, we screened 6484 titles and abstracts and obtained 123 publications for full-text review. Of these, 36 publications (Table 1)^16^^–^^51^ met our eligibility criteria: 16 qualitative studies (Table 2; available from: https://www.who.int/publications/journals/bulletin), ^20^^,^^23^^,^^24^^,^^26^^,^^28^^–^^30^^,^^32^^,^^33^^,^^38^^,^^41^^,^^42^^,^^45^^,^^47^^,^^48^^,^^51^ 18 quantitative studies (Table 3; available from: https://www.who.int/publications/journals/bulletin) ^17^^,^^18^^,^^21^^,^^22^^,^^25^^,^^27^^,^^31^^,^^34^^–^^37^^,^^39^^,^^40^^,^^43^^,^^44^^,^^46^^,^^49^^,^^50^ and two mixed-methods studies^16^^,^^19^ (Table 2, Table 3 and Fig. 1). According to the calculated AHRQ score, six studies were classified as having a high risk of bias^20^^,^^24^^,^^28^^,^^35^^,^^42^^,^^48^ and 30 studies as having a medium risk of bias.^16^^–^^19^^,^^21^^–^^23^^,^^25^^–^^27^^,^^29^^–^^34^^,^^36^^–^^41^^,^^43^^–^^47^^,^^49^^–^^51^

**Table 1:** Characteristics and risk of bias of studies included in systematic review of health workers’ adoption of digital health technology in low- and middle-income countries

Reference	Country	Study population	AHRQ score	Risk of bias
Maarop & Win, 2012^16^	Malaysia	72 medical officers, specialists, medical assistants and radiographers	7	Moderate
Adenuga et al., 2017^17^	Nigeria	252 physicians and nurses	7	Moderate
Beglaryan et al., 2017^18^	Armenia	233 physicians and nurses	7	Moderate
Sezgin et al., 2017^19^	Türkiye	137 physicians	6	Moderate
Damasceno & Caldeira, 2018^20^	Brazil	86 health managers	4	High
Sezgin et al., 2018^21^	Türkiye	122 physicians (general practitioners and specialist medical practitioners)	5	Moderate
Zayyad & Toycan, 2018^22^	Nigeria	465 doctors, nurses, radiologists, laboratory technologists and medical directors	6	Moderate
Damasceno & Caldeira, 2019^23^	Brazil	385 physicians	5	Moderate
Han et al., 2019^24^	Sri Lanka	29 health professionals	1	High
Pan et al., 2019^25^	China	149 non-clinicians (e.g. pathology, radiology, laboratory), 345 clinicians (e.g. surgery, orthopaedics, gastroenterology, neurosurgery)	7	Moderate
Peprah et al., 2020^26^	Ghana	45 health workers	7	Moderate
Pan & Gao, 2021^27^	China	1207 nurses	6	Moderate
Sekandi et al., 2021^28^	Uganda	30 health workers, caregivers and community volunteer workers	3	High
Thomas et al., 2021^29^	India	10 physicians	6	Moderate
Vasconcelos et al., 2021^30^	Brazil	20 nurses, community health agents, coordinators of the primary health care	6	Moderate
Bakshi & Tandon, 2022^31^	India	215 doctors	6	Moderate
Fernandes et al., 2022^32^	Brazil	717 physical therapists	6	Moderate
Hasan et al., 2022^33^	Bangladesh	15 health professionals	5	Moderate
Husin et al., 2022^34^	Malaysia	149 health workers	6	Moderate
Singh & Ravi, 2022^35^	India	224 medical practitioners	4	High
Yu-tong et al., 2022^36^	China	3386 clinical nurses	8	Moderate
Wu et al., 2022^37^	China	393 physicians	6	Moderate
Acero-Torres et al., 2023^38^	Colombia	430 health-care professionals	6	Moderate
Azam et al., 2023^39^	Pakistan	314 doctors and nurses	7	Moderate
Bian et al., 2023^40^	China	12 031 health-care professionals	8	Moderate
Daniel et al., 2023^41^	India	10 primary health centre doctors	6	Moderate
Huang et al., 2023^42^	India	30 physicians	4	High
Kissi et al., 2023^43^	Ghana	543 physicians, physician assistants, nurses, health-care administrators and telehealth service providers	6	Moderate
Walle et al., 2023^44^	Ethiopia	610 health-care professionals	6	Moderate
Xu et al., 2023^45^	China	22 doctors	5	Moderate
Yao et al., 2023^46^	China	1004 clinical-related general practice working in primary care	7	Moderate
Calderon et al., 2024^47^	Philippines	30 primary health workers	6	Moderate
Kachimanga et al., 2024^48^	Malawi	69 community health workers	4	High
Meng & Guo, 2024^49^	China	216 physicians	7	Moderate
Saifullah et al., 2024^50^	Pakistan	518 health-care practitioners	6	Moderate
Thomas et al., 2024^51^	India	11 nurses and cardiologists	5	Moderate

AHRQ: United States Agency for Healthcare Research and Quality.

**Table 2:** Factors affecting health workers’ adoption of digital health technology in low- and middle-income countries: qualitative studies included in systematic review

Study, factor	Factor domain	Outcome	Facilitator or barrier
**Maarop & Win, 2012** ^16^ ^,a^			
Service need	Performance expectancy	Behavioural intention	Facilitator
Perceived usefulness	Performance expectancy	Behavioural intention	Facilitator
Perceived ease of use of teleconsultation technology	Effort expectancy	Behavioural intention	Both
**Sezgin et al., 2017** ^19^ ^,a^			
Information gathering (personal level)	Performance expectancy	Behavioural intention	Facilitator
Communication (personal level)	Performance expectancy	Behavioural intention	Facilitator
Urgency (personal level)	Performance expectancy	Behavioural intention	Facilitator
Accessibility (personal level)	Facilitating conditions	Behavioural intention	Facilitator
Interest in new technologies (personal level)	Attitude	Behavioural intention	Facilitator
Education (personal level)	Performance expectancy	Behavioural intention	Facilitator
Ease of use (personal level)	Effort expectancy	Behavioural intention	Facilitator
Expectations (personal level)	Performance expectancy	Behavioural intention	Facilitator
Social sharing (personal level)	Social influence	Behavioural intention	Facilitator
Leisure time (personal level)	Effort expectancy	Behavioural intention	Facilitator
Compatibility (organizational level)	Facilitating conditions	Behavioural intention	Facilitator
Performance (organizational level)	Performance expectancy	Behavioural intention	Facilitator
Assistance (organizational level)	Social influence	Behavioural intention	Facilitator
Lack of knowledge and interest (personal level)	Attitude	Behavioural intention	Barrier
Software problems (personal level)	Facilitating conditions	Behavioural intention	Barrier
Anxiety (personal level)	Self-efficacy	Behavioural intention	Barrier
Lack of investment (organizational level)	Facilitating conditions	Behavioural intention	Facilitator
Lack of control (organizational level)	Facilitating conditions	Behavioural intention	Facilitator
Habits (organizational level)	Habit	Behavioural intention	Both
**Damasceno & Caldeira, 2019** ^20^			
Inadequate infrastructure	Facilitating conditions	Actual use	Barrier
Intrinsic motivation	Attitude	Actual use	Barrier
**Damasceno et al., 2019** ^23^			
Unavailability of internet connection at health-care facility	Facilitating conditions	Actual use	Barrier
Lack of information about teleconsulting service	Social influence	Actual use	Barrier
Lack of training for use of teleconsulting service	Facilitating conditions	Actual use	Barrier
**Han et al., 2019** ^24^			
Better service	Performance expectancy	Actual use	Facilitator
Efficiency	Performance expectancy	Actual use	Facilitator
Indirectness of communication	Effort expectancy	Actual use	Barrier
Poverty	Incentive	Actual use	Barrier
Inequality between private and public sectors	Risk	Actual use	Barrier
**Peprah et al., 2020** ^26^			
Reduced issues of cost and transportation	Performance expectancy	Behavioural intention	Facilitator
**Sekandi et al., 2021** ^28^			
Easy monitoring of medication adherence	Performance expectancy	Actual use	Facilitator
Improved communication between patient and provider	Performance expectancy	Actual use	Facilitator
Saved money and time	Performance expectancy	Actual use	Facilitator
Limited technology usability skills	Facilitating conditions	Actual use	Barrier
Inadequate technical infrastructure	Facilitating conditions	Actual use	Barrier
Mobile phone use and skills	Facilitating conditions	Actual use	Barrier
**Thomas et al., 2021** ^29^			
Patients benefitting from subsequent reduction in required clinic visits	Performance expectancy	Actual use	Facilitator
Decreased workload	Performance expectancy	Actual use	Facilitator
Increased job satisfaction	Performance expectancy	Actual use	Facilitator
Less stigmatizing for patients	Performance expectancy	Actual use	Facilitator
Intermittent (every 72 hours) updating of patients’ adherence records	Performance expectancy	Actual use	Barrier
Digital organization and labelling of medications	Effort expectancy	Actual use	Facilitator
Training in use of medication event reminder monitor	Facilitating conditions	Actual use	Facilitator
**Vasconcelos et al., 2021** ^30^			
Technological anxiety	Self-efficacy	Behavioural intention	Barrier
**Fernandesa et al., 2022** ^32^			
Data privacy	Risk	Actual use	Barrier
Adequate infrastructure^b^	Facilitating conditions	Actual use	Facilitator
**Hasan et al., 2022** ^33^			
Economic cost	Incentive	Behavioural intention	Both
Social influence by culture and family support	Social influence	Behavioural intention	Facilitator
Perceived enjoyment using the technology	Attitude	Behavioural intention	Facilitator
Facilitating conditions as a tool for promoting patients’ confidence about structural, environmental and process resources	Facilitating conditions	Behavioural intention	Facilitator
Training on the appropriate and efficient usage of mHealth	Facilitating conditions	Behavioural intention	Facilitator
Reward	Incentive	Behavioural intention	Facilitator
**Acero-Torres et al., 2023** ^38^			
Difficulty of use	Effort expectancy	Actual use	Barrier
**Daniel et al., 2023** ^41^			
Technical challenges	Effort expectancy	Actual use	Barrier
**Huang et al., 2023** ^42^			
Perceived usefulness of AI-enabled CDSS	Performance expectancy	Actual use	Facilitator
Perceived impairment of clinical judgement by AI-enabled CDSS	Performance expectancy	Actual use	Facilitator
Perceived impediment of work efficiency by AI-enabled CDSS	Performance expectancy	Actual use	Facilitator
Achieving familiarization with a new system	Effort expectancy	Actual use	Facilitator
Time required to use the system	Effort expectancy	Actual use	Facilitator
Influence of professional hierarchy in decision-making in antibiotic prescribing	Social influence	Actual use	Facilitator
Validated and up-to-date algorithms	Facilitating conditions	Actual use	Facilitator
Workflow integration	Facilitating conditions	Actual use	Facilitator
IT infrastructure	Facilitating conditions	Actual use	Facilitator
Training and technical support	Facilitating conditions	Actual use	Facilitator
Co-creation	Facilitating conditions	Actual use	Facilitator
Cost–effectiveness considerations	Facilitating conditions	Actual use	Facilitator
**Xu et al., 2023** ^45^			
Financial incentive	Incentive	Actual use	Facilitator
Reduction in repetitive and inefficient tasks	Effort expectancy	Actual use	Facilitator
Too busy to use	Risk	Actual use	Barrier
Clinical departments	Facilitating conditions	Actual use	Both
Managerial positions	Facilitating conditions	Actual use	Barrier
Underlying attitudes at affiliated public hospitals	Facilitating conditions	Actual use	Facilitator
Quality management of third-party platforms	Facilitating conditions	Actual use	Facilitator
**Calderon et al., 2024** ^47^			
Internet access	Facilitating conditions	Actual use	Facilitator
Length of time to download the application	Facilitating conditions	Actual use	Barrier
Electricity sources	Facilitating conditions	Actual use	Facilitator
Smartphone	Facilitating conditions	Actual use	Facilitator
Language	Facilitating conditions	Actual use	Facilitator
Organizational structure of the primary care workplace	Facilitating conditions	Actual use	Both
Ease of use and compatibility with existing workflow	Effort expectancy	Actual use	Facilitator
Empowered clinical decision-making	Performance expectancy	Actual use	Facilitator
**Kachimanga et al., 2024** ^48^			
Inadequate data and network connectivity	Facilitating conditions	Actual use	Barrier
Trust	Trust	Actual use	Facilitator
Perceived ease of use	Performance expectancy	Actual use	Facilitator
**Thomas et al., 2024** ^51^			
Lack of training and confidence	Facilitating conditions	Behavioural intention	Barrier

AI: artificial intelligence; CDSS: clinical decision support system; IT: information technology; mHealth: mobile health.

^a^ The studies are of a mixed-methods design.

^b^ Include computer or smartphone for videoconferencing, enough physical space, good internet connection, adequate digital literacy skills.

**Table 3:** Factors affecting health workers’ adoption of digital health technology in low- and middle-income countries: quantitative studies included in systematic review

Study, factors	Factor domain	Outcome	Direction	Effect estimation
**Maarop & Win, 2012** ^16^ **^,a^**				
Service need	Performance expectancy	Behavioural intention	Facilitator	0.552^b^
Perceived usefulness	Performance expectancy	Behavioural intention	Facilitator	0.428^b^
Perceived ease of use	Effort expectancy	Behavioural intention	Facilitator	0.205^b^
**Adenuga et al., 2017** ^17^				
NR	Performance expectancy	Behavioural intention	Facilitator	0.090
NR	Effort expectancy	Behavioural intention	Facilitator	0.122
NR	Facilitating conditions	Behavioural intention	Facilitator	0.165
NR	Social influence	Behavioural intention	Barrier	−0.090
Reinforcement factor	Incentive	Behavioural intention	Facilitator	0.620
**Beglaryan et al., 2017** ^18^				
Personal innovativeness	Self-efficacy	Behavioural intention	Facilitator	0.325
Computer anxiety	Self-efficacy	Behavioural intention	Facilitator	0.019
Patient influence	Performance expectancy	Behavioural intention	Barrier	−0.269
Organizational support	Facilitating conditions	Behavioural intention	Facilitator	0.053
Organizational change	Effort expectancy	Behavioural intention	Barrier	−0.147
Projected collective usefulness	Performance expectancy	Behavioural intention	Facilitator	0.559
**Sezgin et al., 2017** ^19^ ^,a^				
NR	Performance expectancy	Behavioural intention	Facilitator	0.359
NR	Effort expectancy	Behavioural intention	Facilitator	0.106
NR	Social influence	Behavioural intention	Facilitator	0.063
NR	Habit	Behavioural intention	Facilitator	0.077
Technical support and training	Facilitating conditions	Behavioural intention	Barrier	−0.060
Perceived service availability	Effort expectancy	Behavioural intention	Facilitator	0.120
Personal innovativeness	Self-efficacy	Behavioural intention	Facilitator	0.139
Compatibility	Facilitating conditions	Behavioural intention	Barrier	−0.105
Computer self-efficacy	Self-efficacy	Behavioural intention	Facilitator	0.118
Computer anxiety	Self-efficacy	Behavioural intention	Barrier	−0.160
**Sezgin et al., 2018** ^21^				
NR	Performance expectancy	Behavioural intention	Facilitator	0.025
NR	Social influence	Behavioural intention	Barrier	−0.095
NR	Effort expectancy	Behavioural intention	Facilitator	0.215
Compatibility	Facilitating conditions	Behavioural intention	Facilitator	0.189
Technical support and training	Facilitating conditions	Behavioural intention	Barrier	−0.182
Perceived service availability	Effort expectancy	Behavioural intention	Facilitator	0.409
NR	Habit	Behavioural intention	Facilitator	0.061
Mobile anxiety	Self-efficacy	Behavioural intention	Barrier	−0.105
Mobile self-efficacy	Self-efficacy	Behavioural intention	Facilitator	0.129
Personal innovativeness	Self-efficacy	Behavioural intention	Barrier	−0.081
**Zayyad & Toycan, 2018** ^22^				
NR	Attitude	Behavioural intention	Facilitator	0.340^b^
Perceived usefulness	Performance expectancy	Behavioural intention	Facilitator	0.380^b^
Technical infrastructures	Facilitating conditions	Behavioural intention	Facilitator	0.350^b^
Security concerns	Risk	Behavioural intention	Facilitator	0.090^b^
**Pan et al., 2019** ^25^ ^,c^				
NR	Attitude	Behavioural intention	Facilitator	0.335
Perceived usefulness	Performance expectancy	Behavioural intention	Facilitator	0.164
Subjective norm	Social influence	Behavioural intention	Facilitator	0.063
Experience of using mHealth	Self-efficacy	Behavioural intention	Facilitator	0.553
NR	Attitude	Behavioural intention	Facilitator	0.254
Perceived usefulness	Performance expectancy	Behavioural intention	Facilitator	0.145
Subjective norm	Social influence	Behavioural intention	Facilitator	0.094
Experience of using mHealth	Self-efficacy	Behavioural intention	Facilitator	0.675
**Pan & Gao, 2021** ^27^				
NR	Performance expectancy	Behavioural intention	Facilitator	0.259
NR	Effort expectancy	Behavioural intention	Facilitator	0.003
NR	Social influence	Behavioural intention	Facilitator	0.296
NR	Facilitating conditions	Behavioural intention	Facilitator	0.063
NR	Risk	Behavioural intention	Barrier	−0.002
NR	Self-efficacy	Behavioural intention	Facilitator	0.344
Perceived incentives	Incentive	Behavioural intention	Facilitator	0.091
**Bakshi & Tandon, 2022** ^31^				
Financial risk	Incentive	Behavioural intention	Barrier	−0.074
Social risk	Risk	Behavioural intention	Barrier	−0.217
Time risk	Risk	Behavioural intention	Barrier	−0.163
Technology risk	Risk	Behavioural intention	Barrier	−0.120
Security and privacy risk	Risk	Behavioural intention	Barrier	−0.124
**Husin et al., 2022** ^34^				
Perceived usefulness	Performance expectancy	Behavioural intention	Facilitator	0.847
Perceived ease of use	Effort expectancy	Behavioural intention	Facilitator	0.162
**Singh & Ravi, 2022** ^35^				
Performance expectancy	Performance expectancy	Behavioural intention	Barrier	−0.166
Attitude	Attitude	Behavioural intention	Facilitator	0.374
**Yu-tong et al., 2022** ^36^				
Mode cognition	Self-efficacy	Behavioural intention	Facilitator	0.111
Service experience	Self-efficacy	Behavioural intention	Facilitator	0.132
Policy guidance	Facilitating conditions	Behavioural intention	Facilitator	0.104
Manpower allocation	Facilitating conditions	Behavioural intention	Facilitator	0.088
**Wu et al., 2022** ^37^				
NR	Performance expectancy	Behavioural intention	Facilitator	0.283
NR	Effort expectancy	Behavioural intention	Facilitator	0.382
NR	Social influence	Behavioural intention	Facilitator	0.308
NR	Facilitating conditions	Behavioural intention	Facilitator	0.339
NR	Facilitating conditions	Actual use	Facilitator	0.441
NR	Habit	Behavioural intention	Facilitator	0.205
Cognitive trust	Trust	Behavioural intention	Facilitator	0.327
Online rating	Facilitating conditions	Behavioural intention	Facilitator	0.148
Online rating	Facilitating conditions	Actual use	Facilitator	0.449
Behaviour intention	Behaviour intention	Actual use	Facilitator	0.605
**Azam et al., 2023** ^39^				
NR	Performance expectancy	Behavioural intention	Facilitator	0.504
NR	Effort expectancy	Behavioural intention	Barrier	−0.198
NR	Social influence	Behavioural intention	Barrier	−0.134
Self-concept	Self-efficacy	Behavioural intention	Facilitator	0.860
NR	Facilitating conditions	Actual use	Facilitator	0.219
NR	Behavioural intention	Actual use	Barrier	−0.008
**Bian et al., 2023** ^40^				
Perceived value	Performance expectancy	Behavioural intention	Facilitator	0.725
**Kissi et al., 2023** ^43^				
Perceived patient security	Risk	Behavioural intention	Facilitator	0.179
Perceived patient privacy	Risk	Behavioural intention	Facilitator	0.172
Perceived telemedicine systems security	Risk	Behavioural intention	Facilitator	0.097
NR	Self-efficacy	Behavioural intention	Facilitator	0.118
Response efficacy	Performance expectancy	Behavioural intention	Facilitator	0.016
Intention to adopt	Behavioural intention	Actual use	Facilitator	0.089
**Walle et al., 2023** ^44^				
Perceived ease of use	Effort expectancy	Behavioural intention	Facilitator	0.377
Perceived usefulness	Performance expectancy	Behavioural intention	Barrier	−0.013
Digital literacy	Self-efficacy	Behavioural intention	Facilitator	0.087
NR	Attitude	Behavioural intention	Facilitator	0.361
**Yao et al., 2023** ^46^				
NR	Performance expectancy	Behavioural intention	Facilitator	0.199
NR	Effort expectancy	Behavioural intention	Barrier	−0.079
NR	Social influence	Behavioural intention	Facilitator	0.403
NR	Facilitating conditions	Behavioural intention	Barrier	−0.014
Perceived risk	Risk	Behavioural intention	Barrier	−0.085
Price perception	Incentive	Behavioural intention	Facilitator	0.585
**Meng & Guo, 2024** ^49^				
NR	Performance expectancy	Behavioural intention	Facilitator	0.152
NR	Effort expectancy	Behavioural intention	Facilitator	0.109
NR	Social influence	Behavioural intention	Facilitator	0.323
NR	Facilitating conditions	Behavioural intention	Facilitator	0.405
Safety	Trust	Behavioural intention	Facilitator	0.631
**Saifullah et al., 2024** ^50^				
Price value	Incentive	Behavioural intention	Facilitator	0.131
Information quality	Performance expectancy	Behavioural intention	Facilitator	0.299
Perceived system effectiveness	Performance expectancy	Behavioural intention	Facilitator	0.199
Safety	Risk	Behavioural intention	Facilitator	0.134
Waiting time	Effort expectancy	Behavioural intention	Facilitator	0.197
NR	Behavioural intention	Actual use	Facilitator	0.637

mHealth: mobile health; NR: not reported.

^a^ The studies are of a mixed-methods design.

^b^ These studies reported correlation coefficients instead of the *β* estimation in the structural equation modelling.

^c^ The study separately estimated the strengths in clinicians and non-clinicians.^25^

Note: some studies only reported the facilitator or barrier in terms of the factor domain.

All studies were published after the year 2012; the increasing number of publications each year highlights the emerging interest in the acceptance and use of digital health technology in low- and middle-income countries. Our reviewed studies were conducted in 16 low- and middle-income countries, namely: Armenia,^18^ Bangladesh,^33^ Brazil,^20^^,^^23^^,^^30^^,^^32^ China,^25^^,^^27^^,^^36^^,^^37^^,^^40^^,^^45^^,^^46^^,^^49^ Colombia,^38^ Ethiopia,^44^ Ghana,^26^^,^^43^ India,^29^^,^^31^^,^^35^^,^^41^^,^^42^^,^^51^ Malawi,^48^ Malaysia,^16^^,^^34^ Nigeria,^17^^,^^22^ Pakistan,^39^^,^^50^ Philippines,^47^ Sri Lanka^24^ Türkiye^19^^,^^21^ and Uganda^28^. Sample size varied from 10^29^^,^^41^ to 717^32^ for qualitative studies, and from 122^21^ to 12 031^40^ for quantitative studies. Most studies were general in nature and did not consider a specific disease or condition; in contrast, some studies focused on cardiovascular disease,^46^ heart failure^51^, mental disorders^41^, antibiotic prescribing^42^ and tuberculosis^28^. Most studies reported on the experiences of health workers (e.g. doctors, nurses, community health workers), although two papers^20^^,^^22^ also considered the viewpoints of health managers and medical directors. One study separately estimated the facilitators and barriers for clinicians and non-clinicians.^25^ With regards to the type of digital health technology, most studies considered a digital health technology or platform; in contrast, one study focused entirely on wearable electrocardiograph devices.^46^

### Barriers and facilitators 

We list facilitators and barriers, classified as one of the 10 factor domains, in Table 2 and Table 3; we also report the relevant outcome on which the facilitator or barrier had an effect. All of the 10 factor domains were reported as facilitators, and all except for trust and habit were also reported as barriers. Several qualitative studies reported how some factors acted as both facilitators and barriers, which depended on the local context.^16^^,^^19^^,^^33^^,^^45^^,^^47^ For example, the study conducted in the Philippines reported how the organizational structure of the primary care workplace facilitated the use of an electronic decision support application in rural areas (because the limited number of physicians meant that nurses were more involved in direct patient care), whereas organizational structure was a barrier to use in urban sites.^47^

We observed that the facilitators of behavioural intention and actual use of digital health technology reported in the highest number of reviewed studies were performance expectancy (21 out of 36 reviewed studies; 58.3%), facilitating conditions (14; 38.9%) and effort expectancy (13; 36.1%; Table 4). We noted that the top three barriers to behavioural intention and actual use were facilitating conditions (10; 27.8%), effort expectancy (6; 16.7%) and risk (6; 16.7%).

**Table 4:** Occurrence of the facilitator and barrier domains in the studies included in a systematic review on health workers’ adoption of digital health technology in low- and middle-income countries

Path	No. of studies (*n* = 36)	%
**Facilitator**		
Performance expectancy		
→ behavioural intention	15^16^^–^^18^^,^^21^^,^^22^^,^^25^^,^^27^^,^^34^^,^^37^^,^^39^^,^^40^^,^^43^^,^^46^^,^^49^^,^^50^	41.7
→ actual use	6^24^^,^^28^^,^^29^^,^^42^^,^^47^^,^^48^	16.7
Facilitating conditions		
→ behavioural intention	7^17^^,^^18^^,^^21^^,^^22^^,^^27^^,^^33^^,^^49^	19.4
→ actual use	7^29^^,^^32^^,^^37^^,^^39^^,^^42^^,^^45^^,^^47^	19.4
Effort expectancy		
→ behavioural intention	9^16^^,^^17^^,^^21^^,^^27^^,^^34^^,^^37^^,^^44^^,^^49^^,^^50^	25.0
→ actual use	4^29^^,^^42^^,^^45^^,^^47^	11.1
Self-efficacy – behavioural intention	8^18^^,^^21^^,^^25^^,^^27^^,^^36^^,^^39^^,^^43^^,^^44^	22.2
Social influence		
→ behavioural intention	7^19^^,^^25^^,^^27^^,^^33^^,^^37^^,^^46^^,^^49^	19.4
→ actual use	1^42^	2.8
Incentive		
→ behavioural intention	5^17^^,^^27^^,^^33^^,^^46^^,^^50^	13.9
→ actual use	1^45^	2.8
Attitude → behavioural intention	5^22^^,^^25^^,^^33^^,^^35^^,^^44^	13.9
Risk → behavioural intention	3^22^^,^^43^^,^^50^	8.3
Trust		
→ behavioural intention	2^37^^,^^49^	5.6
→ actual use	1^48^	2.8
Habit → behavioural intention	2^21^^,^^37^	5.6
Behavioural intention → actual use	3^37^^,^^43^^,^^50^	8.3
**Barrier**		
Facilitating conditions		
→ behavioural intention	4^19^^,^^21^^,^^46^^,^^51^	11.1
→ actual use	6^20^^,^^23^^,^^28^^,^^45^^,^^47^^,^^48^	16.7
Effort expectancy		
→ behavioural intention	3^18^^,^^39^^,^^46^	8.3
→ actual use	3^24^^,^^38^^,^^41^	8.3
Risk		
→ behavioural intention	3^27^^,^^31^^,^^46^	8.3
→ actual use	3^24^^,^^32^^,^^45^	8.3
Performance expectancy		
→ behavioural intention	3^18^^,^^35^^,^^44^	8.3
→ actual use	1^29^	2.8
Social influence		
→ behavioural intention	3^17^^,^^21^^,^^39^	8.3
→ actual use	1^23^	2.8
Incentive		
→ behavioural intention	2^31^^,^^33^	5.6
→ actual use	1^24^	2.8
Self-efficacy → behavioural intention	3^19^^,^^21^^,^^30^	8.3
Attitude → actual use	1^20^	2.8
Behavioural intention → actual use	1^39^	2.8

### Meta-analysis

**Fig. 2:**
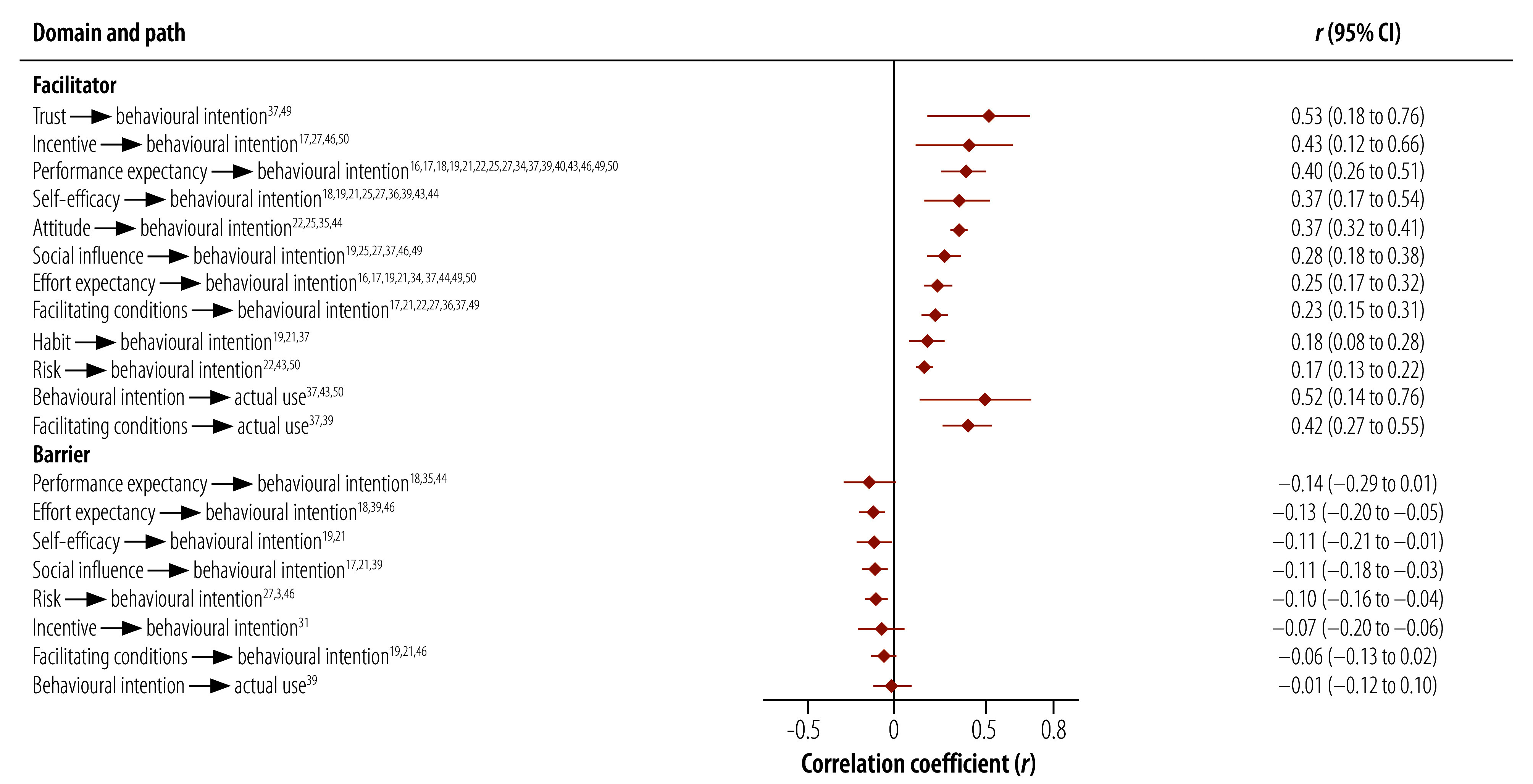
Correlation between facilitators and barriers and use of digital health technology by health workers in low- and middle-income countries

Notes. A full analysis for each path is provided in the online repository.^8^

Our meta-analysis of the correlation coefficient reported in the 18 quantitative and two mixed-methods studies (Table 3) allowed us to quantify the effect of each reported factor on the acceptance and use of the digital technology (Fig. 2 and online repository).^8^ We observed that trust (*r* = 0.53; 95% CI: 0.18 to 0.76) and incentive (*r* = 0.43; 95% CI: 0.12 to 0.66) were the leading facilitators of the behavioural intention to use digital technology, and facilitating conditions (*r* = 0.42; 95% CI: 0.27 to 0.55) was the leading facilitator of its actual use. Concerns with performance expectancy (*r* = −0.14; 95% CI: −0.29 to 0.01), anxiety about effort expectancy (*r* = −0.13; 95% CI: −0.20 to −0.05) and lack of self-efficacy (*r* = −0.11; 95% CI: −0.21 to −0.01) were the primary barriers to behavioural intention to use digital health technologies.

We also estimated the strengths of facilitators and barriers in upper-middle-income counties and in low- and lower-middle-income countries separately (online repository).^8^ We observed heterogeneity between the domains facilitating conditions and risk and the acceptance and use of digital health technologies. In upper-middle-income countries, facilitating conditions were a facilitator to the actual use of digital health technologies (*r* = 0.49 for upper-middle-income countries, compared with *r* = 0.26 for lower-middle- and low-income-countries; *P* < 0.001). In lower-middle- and low-income-countries, concerns with regards to the related risks of digital health formed a strong barrier (*r* = −0.15 for lower-middle- and low-income-countries, compared with *r* = −0.04 for upper-middle-income countries; *P* = 0.035).

We conducted a sensitivity analysis by excluding the single quantitative study with a high risk of bias,^35^ and observed slight changes in only two framework paths (online repository).^8^ We observed that the factor domain of attitude was a facilitator to behavioural intention to use digital health technology (*r* = 0.37; 95% CI: 0.32 to 0.41), and performance expectancy was a barrier (*r* = −0.14; 95% CI: −0.37 to 0.12). We conducted another sensitivity analysis by excluding studies with sample sizes smaller than the median. We observed that the factor domains of trust, performance expectancy and attitude were the leading facilitators of the intention to use digital health technology, and facilitating conditions was the leading facilitator of actual use; self-efficacy remained the greatest barrier to both intention to use and actual use (online repository).^8^

## Discussion

Although the launch of the *Global strategy on digital health 2020–2025*^6^ acknowledged the urgent need to address the issues faced by least-developed countries in their implementation of digital health technologies, our systematic review has highlighted that research remains limited, exacerbating inequity in health digitalization.^52^^,^^53^ Our review highlighted increasing interest in health digitalization particularly in Brazil, China and India, and insufficient focus on this topic in other low- and middle-income countries. A previous scoping review on the facilitators of and barriers to digital health technologies^54^ similarly reported that studies on this topic were concentrated in high-income countries. However, knowledge of facilitators and barriers is essential in the design of digital health programmes for optimized implementation and the attainment of favourable outcomes. Although health workers have been the focus in previous digital health intervention studies,^55^^,^^56^ the limited focus on acceptance and use among these populations reveals a research gap that requires the development of an enabling policy environment.^4^^,^^57^

Facilitating conditions was the most frequently mentioned factor domain in the reviewed studies, and had a strong association with the behavioural intention of health workers. We observed that three tiers of supporting facilities were mentioned in the reviewed studies: infrastructure, technical training and organization management. Infrastructure, such as internet access, electricity sources and information technology, is fundamental for digital health technology. Strengthened supporting facilities could significantly improve the use of digital health technology, as reported in Brazil^32^ and the Philippines,^47^ while inadequate conditions regarding internet connection^23^ and appropriate software^19^ would act as barriers, especially in low-income countries. The availability of technical training on the efficient use of digital health technology was also reported as a significant facilitator, while limited technology skills and a lack of training and confidence were identified as key challenges from the perspective of health workers. A study in China reported on the influence of institutional and organizational factors, such as the clinical departments and attitudes and regulations of the hospitals.^45^

The provision of incentive policies could guide the acceptance and use of digital health technology by health workers. Empirical evidence indicates that financial incentives, such as subsidies for purchasing digital devices, performance-based bonuses and funding for continuous professional development, significantly enhance the propensity of health workers to adopt and integrate these technologies within their practice.^58^ A mixed-methods analysis reported that financial incentives were one of the most important improvement strategies for digital health adoption.^59^ Non-financial incentives also play a pivotal role, for example, opportunities for professional growth, and formal recognition through awards or certifications, significantly enhance motivation to use digital health technology. A study in sub-Saharan African countries indicated that structured training programmes and certification courses for telemedicine platforms significantly increased their uptake among health workers.^60^ The strategic alignment of these incentive structures with the overarching objectives of health workers not only creates a conducive environment for digital health solutions but also fosters sustained engagement and utilization. 

We also observed how personal and psychological factors are key drivers in promoting the adoption of digital health technologies. For instance, health-care professionals’ perceptions of usefulness and their willingness to adapt were frequently cited facilitators. These beliefs could offset concerns and anxieties associated with the technologies, which were identified as major barriers to implementation (especially in low-income countries). Evidence showed that educational activities tailored to the specific needs of health workers, combined with user-friendly designs, intuitive system navigation and easy-to-use interfaces, could effectively address personal concerns. 

Our study had several limitations. By focusing on the perspectives of health workers, the views of other important stakeholders (e.g. health management and support personnel, government officials and representatives of the technology industry) were not considered. Second, we could not rule out the influence of the selective reporting of positive or negative results. Third, although we searched six databases with no language restrictions, potentially relevant studies catalogued elsewhere were not considered.

To conclude, the findings from our study have implications for the development of policies to promote digital health technology in low- and middle-income countries. Our novel approach of clustering the facilitators of and barriers to the acceptance and use of digital health technology from the perspective of health workers highlighted the importance of creating an enabling ecosystem; supportive infrastructure, tailored training programmes and incentive policies should all be incorporated in the implementation of digital health programmes. 

## References

[1] Solomon DH, Rudin RS. Digital health technologies: opportunities and challenges in rheumatology. Nat Rev Rheumatol. 2020 Sep;16(9):525–35. 10.1038/s41584-020-0461-x 32709998

[2] Ndayishimiye C, Lopes H, Middleton J. A systematic scoping review of digital health technologies during COVID-19: a new normal in primary health care delivery. Health Technol (Berl). 2023;13(2):273–84. 10.1007/s12553-023-00725-7 36628261 PMC9816012

[3] Geldsetzer P, Flores S, Wang G, Flores B, Rogers AB, Bunker A, et al. A systematic review of healthcare provider-targeted mobile applications for non-communicable diseases in low- and middle-income countries. NPJ Digit Med. 2022 Jul 19;5(1):99. 10.1038/s41746-022-00644-3 35853936 PMC9296618

[4] Monitoring and evaluating digital health interventions: a practical guide to conducting research and assessment. Geneva: World Health Organization; 2016. Available from: https://iris.who.int/handle/10665/252183 [cited 2024 Nov 11].

[5] Resolution WHA58.28. eHealth. In: Fifty‐eighth World Health Assembly, Geneva, 25 May 2005. Geneva: World Health Organization; 2005. Available from: https://apps.who.int/gb/ebwha/pdf_files/WHA58/WHA58_28-en.pdf [cited 2024 Nov 11].

[6] Global strategy on digital health 2020–2025. Geneva: World Health Organization; 2021. Available from: https://iris.who.int/handle/10665/344249 [cited 2024 Nov 11].

[7] Page MJ, McKenzie JE, Bossuyt PM, Boutron I, Hoffmann TC, Mulrow CD, et al. The PRISMA 2020 statement: an updated guideline for reporting systematic reviews. BMJ. 2021 Mar 29;372(71):n71. 10.1136/bmj.n71 33782057 PMC8005924

[8] Wang M, Huang K, Li X, Zhao X, Downey L, Hassounah S, et al. Health workers’ adoption of digital health technology in low- and middle-income countries: a systematic review. Supplemental file [data repository]. London: figshare; 2024. 10.6084/m9.figshare.27850896 PMC11774224 39882495

[9] Viswanathan M, Ansari MT, Berkman ND, Chang S, Hartling L, McPheeters M, et al. Assessing the risk of bias of individual studies in systematic reviews of health care interventions. In: Norris S, editor. Methods guide for effectiveness and comparative effectiveness reviews. Rockville: United States Agency for Healthcare Research and Quality; 2008. 22479713

[10] Thomas J, Harden A. Methods for the thematic synthesis of qualitative research in systematic reviews. BMC Med Res Methodol. 2008 Jul 10;8(1):45. 10.1186/1471-2288-8-45 18616818 PMC2478656

[11] Chang A. UTAUT and UTAUT 2: a review and agenda for future research. J Winners. 2012;13(2):10–114. 10.21512/tw.v13i2.656

[12] Williams MD, Rana NP, Dwivedi YK. The unified theory of acceptance and use of technology (UTAUT): a literature review. J Enterp Inf Manag. 2015;28(3):443–88. 10.1108/JEIM-09-2014-0088

[13] Higgins JP, Thompson SG, Deeks JJ, Altman DG. Measuring inconsistency in meta-analyses. BMJ. 2003 Sep 6;327(7414):557–60. 10.1136/bmj.327.7414.557 12958120 PMC192859

[14] Egger M, Smith GD, Schneider M, Minder C. Bias in meta-analysis detected by a simple, graphical test. BMJ. 1997 Sep 13;315(7109):629–34. 10.1136/bmj.315.7109.629 9310563 PMC2127453

[15] Begg CB, Mazumdar M. Operating characteristics of a rank correlation test for publication bias. Biometrics. 1994 Dec;50(4):1088–101. 10.2307/2533446 7786990

[16] Maarop N, Win KT. Understanding the need of health care providers for teleconsultation and technological attributes in relation to the acceptance of teleconsultation in Malaysia: a mixed methods study. J Med Syst. 2012 Oct;36(5):2881–92. 10.1007/s10916-011-9766-2 21826500

[17] Adenuga KI, Iahad NA, Miskon S. Towards reinforcing telemedicine adoption amongst clinicians in Nigeria. Int J Med Inform. 2017 Aug;104:84–96. 10.1016/j.ijmedinf.2017.05.008 28599820

[18] Beglaryan M, Petrosyan V, Bunker E. Development of a tripolar model of technology acceptance: hospital-based physicians’ perspective on EHR. Int J Med Inform. 2017 Jun;102:50–61. 10.1016/j.ijmedinf.2017.02.013 28495348

[19] Sezgin E, Özkan-Yildirim S, Yildirim S. Investigation of physicians’ awareness and use of mHealth apps: a mixed method study. Health Policy Technol. 2017;6(3):251–67. 10.1016/j.hlpt.2017.07.007

[20] Damasceno RF, Caldeira AP. Teleconsultoria na atenção primária no norte de Minas Gerais: cenário e fatores associados à não utilização por médicos. Rev Electron Comun Inf Inov Saude. 2018;12(4):456–65. Portuguese. 10.29397/reciis.v12i4.1312

[21] Sezgin E, Özkan-Yildirim S, Yildirim S. Understanding the perception towards using mHealth applications in practice: Physicians’ perspective. Inf Dev. 2018;34(2):182–200. 10.1177/0266666916684180

[22] Zayyad MA, Toycan M. Factors affecting sustainable adoption of e-health technology in developing countries: an exploratory survey of Nigerian hospitals from the perspective of healthcare professionals. PeerJ. 2018 Mar 1;6:e4436. 10.7717/peerj.4436 29507830 PMC5835346

[23] Damasceno RF, Caldeira AP. Fatores associados à não utilização da teleconsultoria por médicos da Estratégia Saúde da Família. Cien Saude Colet. 2019 Aug 5;24(8):3089–98. Portuguese. 10.1590/1413-81232018248.28752017 31389555

[24] Han KJ, Subramanian R, Cameron GT. Listen before you leap: Sri Lankan health professionals’ perspectives on m-health. Health Informatics J. 2019 Sep;25(3):858–66. 10.1177/1460458217725903 28825329

[25] Pan J, Ding S, Wu D, Yang S, Yang J. Exploring behavioural intentions toward smart healthcare services among medical practitioners: a technology transfer perspective. Int J Prod Res. 2019;57(18):5801–20. 10.1080/00207543.2018.1550272

[26] Peprah P, Abalo EM, Agyemang-Duah W, Budu HI, Appiah-Brempong E, Morgan AK, et al. Lessening barriers to healthcare in rural Ghana: providers and users’ perspectives on the role of mHealth technology. A qualitative exploration. BMC Med Inform Decis Mak. 2020 Feb 10;20(1):27. 10.1186/s12911-020-1040-4 32041608 PMC7011292

[27] Pan M, Gao W. Determinants of the behavioral intention to use a mobile nursing application by nurses in China. BMC Health Serv Res. 2021 Mar 12;21(1):228. 10.1186/s12913-021-06244-3 33712012 PMC7953719

[28] Sekandi JN, Kasiita V, Onuoha NA, Zalwango S, Nakkonde D, Kaawa-Mafigiri D, et al. Stakeholders’ perceptions of benefits of and barriers to using video-observed treatment for monitoring patients with tuberculosis in Uganda: exploratory qualitative study. JMIR Mhealth Uhealth. 2021 Oct 27;9(10):e27131. 10.2196/27131 34704961 PMC8581755

[29] Thomas BE, Kumar JV, Periyasamy M, Khandewale AS, Hephzibah Mercy J, Raj EM, et al. Acceptability of the medication event reminder monitor for promoting adherence to multidrug-resistant tuberculosis therapy in two Indian cities: qualitative study of patients and health care providers. J Med Internet Res. 2021 Jun 10;23(6):e23294. 10.2196/23294 34110300 PMC8262665

[30] Vasconcelos DD, Braz PR, Gontijo TL, de Azevedo Guimarães EA, De Oliveira VC, Zacharias FCM, et al. Implantação e utilização de dispositivo móvel na Atenção Primária à Saúde no Brasil. Rev Cuba Inf Cienc Salud. 2021;32(4). Spanish.

[31] Bakshi S, Tandon U. Understanding barriers of telemedicine adoption: a study in North India. Syst Res Behav Sci. 2022;39(1):128–42. 10.1002/sres.2774

[32] Fernandes LG, Oliveira RFF, Barros PM, Fagundes FRC, Soares RJ, Saragiotto BT. Physical therapists and public perceptions of telerehabilitation: an online open survey on acceptability, preferences, and needs. Braz J Phys Ther. 2022 Nov-Dec;26(6):100464. 10.1016/j.bjpt.2022.100464 36410257 PMC9659283

[33] Hasan N, Sultana R, Bao Y. Re-conceptualizing the drivers toward mHealth adoption in a least developing country: a qualitative exploration. SAGE Open. 2022;12(2). 10.1177/21582440221091719

[34] Husin M, Rahman NA, Bujang MA, Ng SW, Juval K, Hwong WY, et al. Translation and validation of the questionnaire on acceptance to telemedicine from the technology acceptance model (tam) for use in Malaysia. BioMed Res Int. 2022 Apr 20;2022:9123887. 10.1155/2022/9123887 35463970 PMC9020140

[35] Singh A, Ravi P. Adoption of E-health platforms by medical practitioners: mediating effect of attitude on E-health platforms usage. Health Mark Q. 2022 Jan-Mar;39(1):61–73. 10.1080/07359683.2021.1995637 34720067

[36] Yu-tong T, Yan Z, Zhen L, Bing X, Qing-Yun C. Telehealth readiness and its influencing factors among Chinese clinical nurses: A cross-sectional study. Nurse Educ Pract. 2022 Jan;58:103278. 10.1016/j.nepr.2021.103278 34954659

[37] Wu P, Zhang R, Luan J, Zhu M. Factors affecting physicians using mobile health applications: an empirical study. BMC Health Serv Res. 2022 Jan 4;22(1):24. 10.1186/s12913-021-07339-7 34983501 PMC8729011

[38] Acero-Torres DC, Sánchez-Casas YC, Casas-Duarte JP, Páez-Rojas PL, Sánchez-Calderón D, Robayo-González CX, et al. Conocimientos, habilidades, actitudes y prácticas en telesalud de los profesionales de la salud durante la pandemia de COVID-19. Rev Cuba Inf Cienc Salud. 2023;34:e2319. Spanish.

[39] Azam M, Bin Naeem S, Kamel Boulos MN, Faiola A. Modelling the predictors of mobile health (mHealth) adoption among healthcare professionals in low-resource environments. Int J Environ Res Public Health. 2023 Nov 26;20(23):7112. 10.3390/ijerph20237112 38063542 PMC10706785

[40] Bian D, Xiao Y, Song K, Dong M, Li L, Millar R, et al. Determinants influencing the adoption of internet health care technology among Chinese health care professionals: extension of the value-based adoption model with burnout theory. J Med Internet Res. 2023 Mar 10;25:e37671. 10.2196/37671 36897630 PMC10039406

[41] Daniel M, Kaur A, Mukherjee A, Bhattacharya A, Tewari A, Sagar R, et al. The systematic medical appraisal, referral and treatment (SMART) mental health programme: formative research informing a cluster randomized controlled trial. SSM Ment Health. 2023;3:100223. 10.1016/j.ssmmh.2023.100223

[42] Huang Z, George MM, Tan YR, Natarajan K, Devasagayam E, Tay E, et al. Are physicians ready for precision antibiotic prescribing? A qualitative analysis of the acceptance of artificial intelligence-enabled clinical decision support systems in India and Singapore. J Glob Antimicrob Resist. 2023 Dec;35:76–85. 10.1016/j.jgar.2023.08.016 37640155 PMC10684720

[43] Kissi J, Dai B, Achmpong EK, Dankyi AB, Antwi J. An empirical study of healthcare professionals’ willingness to utilise telehealth services based on protection motivation theory. Int J Healthc Technol Manag. 2023;20(1):74–89. 10.1504/IJHTM.2023.130314

[44] Walle AD, Ferede TA, Baykemagn ND, Shimie AW, Kebede SD, Tegegne MD, et al. Predicting healthcare professionals’ acceptance towards electronic personal health record systems in a resource-limited setting: using modified technology acceptance model. BMJ Health Care Inform. 2023 Mar;30(1):e100707. 10.1136/bmjhci-2022-100707 36878620 PMC9990677

[45] Xu D, Huang Y, Tsuei S, Fu H, Yip W. Factors influencing engagement in online dual practice by public hospital doctors in three large cities: a mixed-methods study in China. J Glob Health. 2023 Sep 22;13:04103. 10.7189/jogh.13.04103 37736850 PMC10514738

[46] Yao Y, Li Z, He Y, Zhang Y, Guo Z, Lei Y, et al. Factors affecting wearable ECG device adoption by general practitioners for atrial fibrillation screening: cross-sectional study. Front Public Health. 2023 May 5;11:1128127. 10.3389/fpubh.2023.1128127 37213597 PMC10196261

[47] Calderon Y, Sandigan G, Tan-Lim CSC, De Mesa RYH, Fabian NMC, Rey MP, et al. Feasibility, acceptability and impact of a clinical decision support tool among primary care providers in an urban, rural and remote site in the Philippines. BMJ Open Qual. 2024 Feb 29;13(1):e002526. 10.1136/bmjoq-2023-002526 38423587 PMC10910488

[48] Kachimanga C, Mulwafu M, Ndambo MK, Harare J, Murkherjee J, Kulinkina AV, et al. Experiences of community health workers on adopting mHealth in rural Malawi: a qualitative study. Digit Health. 2024 May 15;10:20552076241253994. 10.1177/20552076241253994 38757088 PMC11097726

[49] Meng D, Guo Z. Influence of doctor-patient trust on the adoption of mobile medical applications during the epidemic: a UTAUT-based analysis. Front Public Health. 2024 Aug 19;12:1414125. 10.3389/fpubh.2024.1414125 39224557 PMC11366569

[50] Saifullah, Ma Z, Li M, Maqbool MQ, Chen J. Enhancing telehealth services development in Pakistani healthcare sectors through examining various medical service quality characteristics. Front Public Health. 2024 Jul 9;12:1376534. 10.3389/fpubh.2024.1376534 39045155 PMC11263101

[51] Thomas SC, Neenumol K, Chacko S, Prinu J, Pillai MR, Pisharody S, et al. Feasibility of a nurse-led, mHealth-assisted, and team-based collaborative care model for heart failure care in India: findings from a multi-stakeholder qualitative study. Wellcome Open Res. 2024 Oct 15;9:219. 10.12688/wellcomeopenres.21175.1 39211807 PMC11358683

[52] Hui CY, Abdulla A, Ahmed Z, Goel H, Monsur Habib GM, Teck Hock T, et al. RESPIRE Group. Mapping national information and communication technology (ICT) infrastructure to the requirements of potential digital health interventions in low- and middle-income countries. J Glob Health. 2022 Dec 29;12:04094. 10.7189/jogh.12.04094 36579436 PMC9804211

[53] Alnasser Y, Proaño A, Loock C, Chuo J, Gilman RH. Telemedicine and pediatric care in rural and remote areas of middle-and-low-income countries: narrative review. J Epidemiol Glob Health. 2024 Sep;14(3):779–86. 10.1007/s44197-024-00214-8 38478166 PMC11442723

[54] Borges do Nascimento IJ, Abdulazeem H, Vasanthan LT, Martinez EZ, Zucoloto ML, Østengaard L, et al. Barriers and facilitators to utilizing digital health technologies by healthcare professionals. NPJ Digit Med. 2023 Sep 18;6(1):161. 10.1038/s41746-023-00899-4 37723240 PMC10507089

[55] Palacholla RS, Fischer N, Coleman A, Agboola S, Kirley K, Felsted J, et al. Provider- and patient-related barriers to and facilitators of digital health technology adoption for hypertension management: scoping review. JMIR Cardio. 2019 Mar 26;3(1):e11951. 10.2196/11951 31758771 PMC6834226

[56] Xiong S, Lu H, Peoples N, Duman EK, Najarro A, Ni Z, et al. Digital health interventions for non-communicable disease management in primary health care in low-and middle-income countries. NPJ Digit Med. 2023 Feb 1;6(1):12. 10.1038/s41746-023-00764-4 36725977 PMC9889958

[57] Wannheden C, Åberg-Wennerholm M, Dahlberg M, Revenäs Å, Tolf S, Eftimovska E, et al. Digital health technologies enabling partnerships in chronic care management: scoping review. J Med Internet Res. 2022 Aug 1;24(8):e38980. 10.2196/38980 35916720 PMC9379797

[58] Allen J, Carbo-Valverde S, Chakravorti S, Rodriguez-Fernandez F, Pinar Ardic O. Assessing incentives to increase digital payment acceptance and usage: a machine learning approach. PLoS One. 2022 Nov 2;17(11):e0276203. 10.1371/journal.pone.0276203 36322584 PMC9629583

[59] Weik L, Fehring L, Mortsiefer A, Meister S. Understanding inherent influencing factors to digital health adoption in general practices through a mixed-methods analysis. NPJ Digit Med. 2024 Feb 27;7(1):47. 10.1038/s41746-024-01049-0 38413767 PMC10899241

[60] Dodoo JE, Al-Samarraie H, Alsswey A. The development of telemedicine programs in Sub-Saharan Africa: progress and associated challenges. Health Technol (Berl). 2022;12(1):33–46. 10.1007/s12553-021-00626-7 34849325 PMC8613515

